# Complicated Hereditary Spastic Paraplegia Caused by *SERAC1* Variants in a Chinese Family

**DOI:** 10.3389/fped.2021.816265

**Published:** 2022-02-11

**Authors:** Dandan Yan, Shaopei Chen, Fengying Cai, Jianbo Shu, Xiufang Zhi, Jie Zheng, Chunhua Zhang, Dong Li, Chunquan Cai

**Affiliations:** ^1^Tianjin Pediatric Research Institute, Tianjin Children's Hospital (Tianjin University Children's Hospital), Tianjin, China; ^2^Tianjin Key Laboratory of Birth Defects for Prevention and Treatment, Tianjin, China; ^3^Department of Neurology, Tianjin Children's Hospital (Tianjin University Children's Hospital), Tianjin, China; ^4^Department of Physiology, Tianjin Medical College, Tianjin, China; ^5^Matsumoto Institute of Life Science (MILS) International, Yokohama, Japan

**Keywords:** *SERAC1*, complicated hereditary spastic paraplegia, novel variant, MEGDEL syndrome, 3-methylglutaconic aciduria

## Abstract

**Background:**

The serine active site-containing protein 1 (*SERAC1*) biallelic variant usually causes MEGDEL syndrome, clinically characterized by increased excretion of 3-methylglutaconic in the urine, muscle hypotonia, sensorineural deafness, and Leigh-like lesions on brain MRI scans. In this study, we present a case from a Chinese family with disordered metabolism and dystonia owing to *SERAC1* variants; the clinical phenotypes of the proband were different from those of MEGDEL syndrome but were similar to those juvenile-onset complicated hereditary spastic paraplegia. Thus, in this study, we aimed to confirm the relationship between *SERAC1* variants and complicated hereditary spastic paraplegia.

**Methods:**

MRI and laboratory tests, including gas chromatography/mass spectrometry (GC/MS), were carried out for the proband. Whole-exome sequencing was used to detect the candidate *SERAC1* variants. Variants were verified using Sanger sequencing. Various software programs (PolyPhen-2, MutationTaster, PROVEAN, and SIFT) were used to predict the pathogenicity of novel variants.

**Results:**

Brain MRI scans showed a symmetric flake abnormal signal shadow in the bilateral basal ganglia in T2-weighted image (T2WI) and fluid-attenuated inversion recovery (FLAIR) analyses. The excretion of 3-methylglutaconic acid was found to be increased in our GC/MS analysis. Whole-exome sequencing showed novel compound heterozygous variants, including a novel c.1495A>G (p.Met499Val) variant in exon 14 of *SERAC1* inherited from the father and a novel c.721_722delAG (p.Leu242fs) variant in exon 8 inherited from the mother. The pathogenicity prediction results showed that these two variants were deleterious.

**Conclusions:**

This study presented a patient with complicated hereditary spastic paraplegia caused by *SERAC1* variants. These findings expand the number of known *SERAC1* variants and the phenotypic spectrum associated with SERAC1 deficiency. This study may contribute to counseling and prevention of hereditary diseases through prenatal.

## Introduction

Usually, an increase in 3-methylglutaconic acid (3-MGA) indicates 3-methylglutaconic aciduria (3-MGA-uria), which was first reported in 2006 by Wortmann and colleagues (1). 3-MGA-uria is a heterogeneous group of metabolic disorders, biochemically characterized by elevated 3-MGA and 3-methylglutaric acid excretion in urine. High uric 3-MGA is a major and rather common clinical characteristic of patients with suspected metabolic disorders, for which it is a phenotypic hallmark, and the key to diagnosis (2). In 2013, Wortmann and colleagues systematically classified 3-MGA-uria into five types (I–V) (3). According to this classification, type I refers to primary 3-MGA-uria, owing to the known pathological mechanism of 3-methylglutaconyl-CoA hydratase deficiency (4). Types unrelated to leucine metabolism are collectively referred to as secondary 3-MGA-uria (types II–V), among which types include type II, III, and V well-defined. However, type IV represents a confusing and ever-growing subgroup encompassing all “unclassified” symptoms (2, 5), which includes MEDGEL syndrome, characterized by 3-MGA-uria, dystonia-deafness, encephalopathy, and Leigh-like syndrome (MEGDEL, OMIM ID: 614739), known to be caused by serine active site containing 1 (*SERAC1*) variants.

MEGDEL syndrome is an autosomal-recessive disorder that presents clinically as 3-MGA-uria, feeding problems, liver failure, spasticity, dystonia, hearing loss, truncal hypotonia, and premature death, usually occurring between 10 and 20 years of age. Wortmann and colleagues reported several patients with 3-MGA-uria and MEGDEL syndrome in 2006 (1). To date, increasing evidence has confirmed the relationship between MEGDEL syndrome and *SERAC1* variants (6–8). However, *SERAC1* variants have been found to cause juvenile-onset complicated hereditary spastic paraplegia (cHSP) as well (9). Clinically, cHSP is a complicated condition, with predominant lower limb spasticity and additional neurological features; it can be inherited in autosomal dominant, autosomal recessive, or X-linked patterns (10). However, the mechanism has not yet been fully elucidated.

Here, we present a child with dyskinesia in both lower limbs, high uric 3-MGA, enhanced muscular tension and tendon reflexes, pyramidal tract injury, and abnormal MRI results, in whom we investigated *SERAC1* variants by whole-exome sequencing (WES).

## Materials and Methods

### Study Proband

The individual included in this study was a 7-year-old female child admitted to Tianjin Children's Hospital in November 2020.

All procedures in this study were performed as per the ethical standards of the institutional review board and followed the Declaration of Helsinki. This study was approved by the Tianjin Children's Hospital Ethics Committee (Reference number 2016021). Written informed consent was obtained from the parents of the proband.

### Genomic DNA Extraction

Genomic DNA was extracted from a whole peripheral blood sample using a Blood Genomic DNA Mini Kit (CWBio, Beijing, China) according to the manufacturer's instructions. The volume of DNA was approximately 100 μl, and the concentration of DNA was >10 ng/μl. The DNA was stored at −20°C until the analyses were performed.

### Genomic Analysis

WES was performed using DNA samples from the proband and both of her parents. We used the Hg 19 human reference genome sequence, along with the Genome Analysis Tool Kit (GATK) software, Annovar, Thousand Human Genome Database, dbSNP, and OMIM, to obtain and notate the clinically relevant genomic variants data. To determine the variant sites, Sanger sequencing was performed on DNA samples from the proband and both of her parents. Chromas software was used to align the sequencing data using reference sequences from GenBank (NM_032861.3).

### Bioinformatics Analysis

The online programs PolyPhen-2, MutationTaster, PROVEAN, and SIFT were used to predict the effects of genetic variants on the functions of *SERAC1*. Furthermore, FASTA-formatted amino acid sequences for *SERAC1* were downloaded from the online program UniProt, and the conservation of the affected amino acids among ten species was analyzed using DNAMAN software.

## Results

### Clinical Analysis

The individual this case study was focused on was born at full term to a gravida 2, para 1, aborta 1, 27-year-old woman by cesarean section and was assigned female at birth. Both parents were healthy at the time of this study, were non-consanguineous, and did not have phenotypic disease characteristics. Physical examinations of the mother were found to be normal during pregnancy. The child had no postnatal asphyxia and achieved developmental milestones at the appropriate age. She had no history of convulsions, abnormal intelligence, or special genetic metabolic.

At the time of our study, the 7-year-old female child was hospitalized due to dyskinesia in the lower left limb for more than 3 years and motor disturbance in the lower right limb for longer than 1 month. Her weight was 16.8 kg (−3 SD to −2 SD), height 118 cm (−1 SD), and cranial circumference 49 cm. When a detailed medical history was taken, we found that the dyskinesia in the lower left limb fluctuated. When the dyskinesia was severe, the lower-left limb was limp when walking; she was able to stand, squat, and go up and down stairs independently, but she could not run or jump. When the dyskinesia was mild, there was no obvious limpness in the lower left limb, and she could run slowly and jump on both legs. One month before her admission, she had a severe limp in her lower right extremity and had myasthenia in both lower extremities while walking, but her upper limbs could move freely.

**Figure 1:**
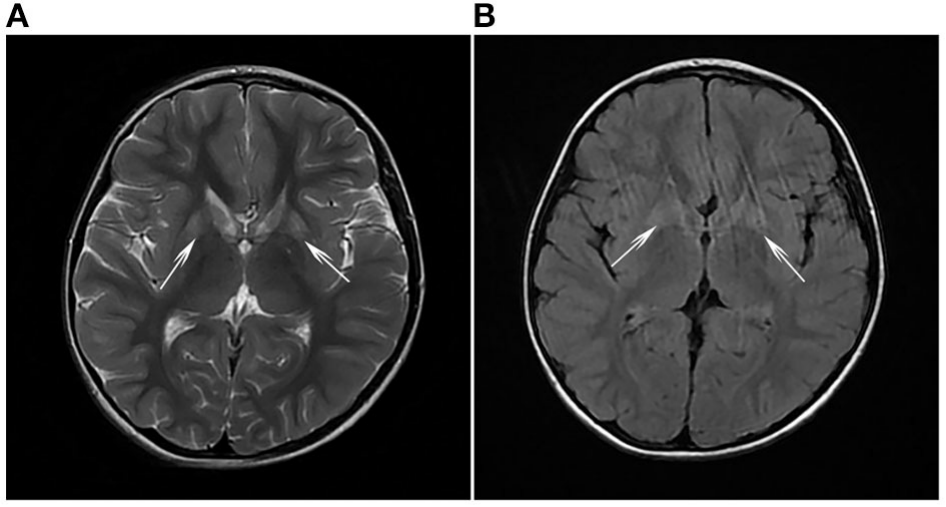
**(A)** T2-weighted imaging (T2WI) and **(B)** fluid-attenuated inversion recovery (FLAIR) analyses revealed symmetrical flake abnormal signal shadows in bilateral basal ganglia (white arrows).

**Figure 2:**
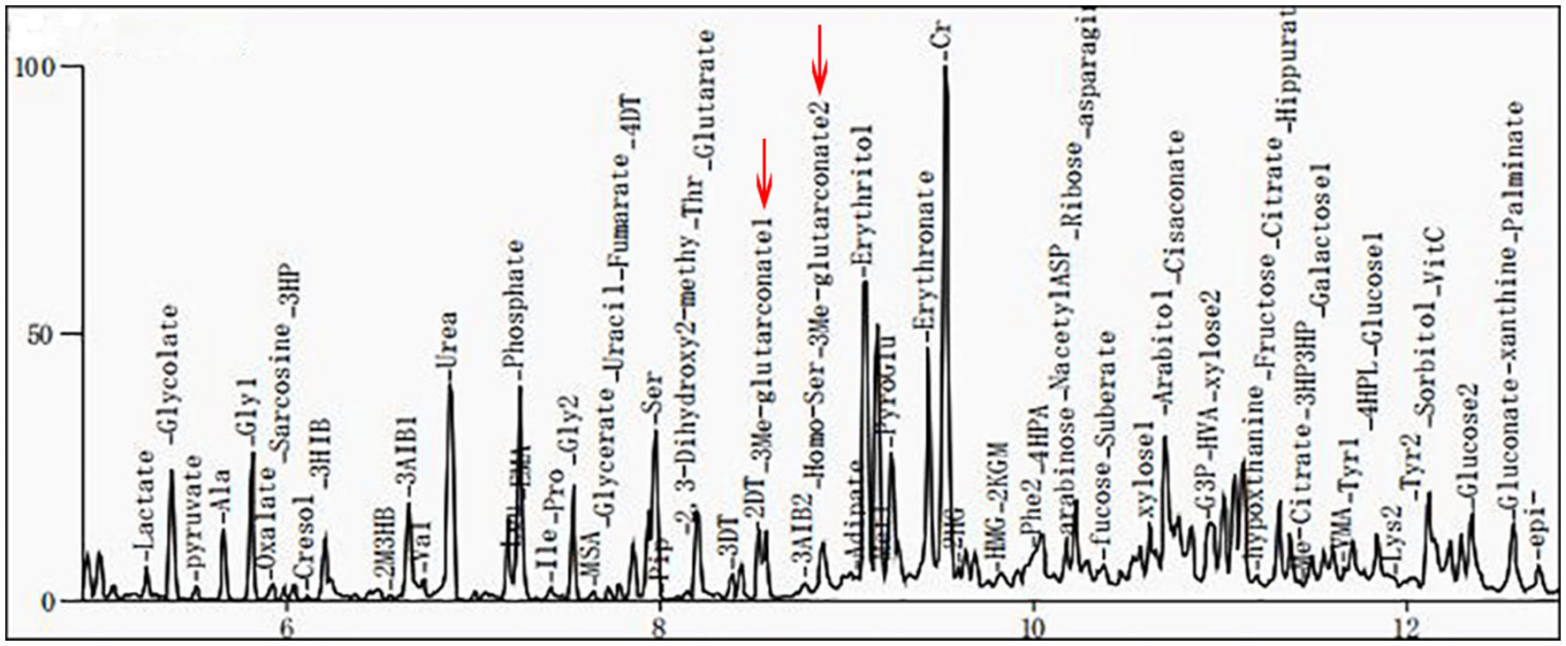
Gas chromatography/mass spectrometry (GC/MS) spectra for 3-methylglutaconic acid (3-MGA) and 3-methylglutaric acid. Results show that urinary excretion of 3-methylglutaric acid and 3-methylglutaric acid were increased (red arrows).

Clinical examination revealed that the muscle tone in the limbs was obviously increased. The myodynamia in both lower extremities was decreased, and the tendon reflexes were hyperactive. An ankle-clonus test was positive. All examination indicators of liver function that were examined were approximately normal. The levels of serum albumin (ALB), alanine aminotransferase (ALT), and aspartate transaminase (AST) were 45 g/L, 11 U/L, and 22 U/L, respectively, within the reference ranges of 38–54 g/L, 7–40 U/L, and 13–35 U/L, respectively. Laboratory tests in this period of time revealed mildly elevated levels of serum lactate (2.32 mmol/L, normal 0.5–2.2 mmol/L) and ammonia (92 μg/dl, normal 12–66 μg/dl). Cholesterol and triglycerides were normal. There were no abnormalities in electroneurogram or electromyography (EMG) results for both lower limbs. Eye fundus examinations were normal with no optic atrophy. Both pupils were equi-circular (diameter = 3 mm) and sensitive to light reflection; both eyeballs were able to move freely with no tremor. No hearing loss was noticed by the patient. Spinal and brain MRI scans of the patient revealed abnormalities in different sections. Spinal MRI scans showed a long T2 signal shadow located at the level of the 6–7th vertebrae. A T2-weighted image (T2WI) showed symmetric flake abnormal signal shadow in the bilateral basal ganglia and slightly widened ventricles (Figure 1A). Fluid-attenuated inversion recovery (FLAIR) analysis of the bilateral basal ganglia revealed a signal intensity that may indicate the involvement of white matter (Figure 1B). Organic acid analysis performed by gas chromatography/mass spectrometry (GC/MS) demonstrated increased excretion of 3-MGA in the urine (Figure 2).

### Genomic Analysis

**Figure 3:**
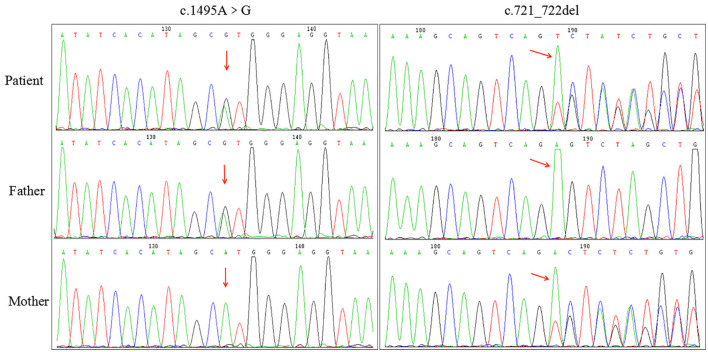
Confirmation of the novel serine active site domain-containing protein (*SERAC1*) variants in the proband and her parents by Sanger sequencing. The proband had compound heterozygous variants in the *SERAC1*: c.1495A>G (p.Met499Val) in exon 14 inherited from her father and c.721_722del (p.Leu242fs) in exon 8 inherited from her mother, resulting in a premature stop codon and a truncated protein (red arrows).

WES revealed novel compound heterozygous variants in *SERAC1* (NM_032861.3) in the proband, including a frameshift variant (c.721_722delAG resulting in Leu242fs) and a missense variant (c.1495A>G resulting in Met499Val). These variants were located on exons 8 and 14. Both variants were not found to be recorded in the Human Gene Mutation Database (HGMD), ESP6500siv2_ALL, 1000 Genomes project, and dbSNP147 database. Sanger sequencing confirmed that the variant c.721_722delAG was inherited from her healthy mother, and the variant c.1495A>G was inherited from her healthy father (Figure 3), both of whom were healthy at the time of this study.

### Bioinformatics Analysis

**Figure 4:**
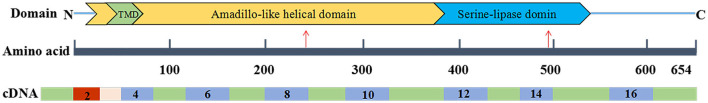
Serine active site domain-containing protein 1 (*SERAC1*) domain. The c.1495A>G variant (p.Met499Val) was located within the serine-lipase domain, and the c.721_722del variant (p.Leu242fs) was located upstream of the serine-lipase domain (indicated by the red arrows).

**Figure 5:**
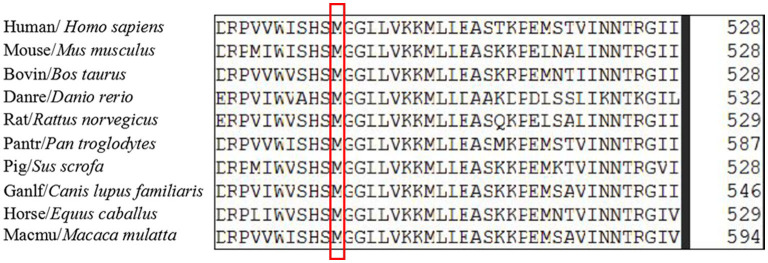
The amino acid sequences alignment of serine active site domain-containing protein (*SERAC1*) homologs among different species was highly conserved at position 499.

The c.721_722del variant was found to be located in the upstream region of the serine-lipase domain, and the c.1495A>G variant was located within the serine-lipase domain (Figure 4). Prediction software analysis showed that the two variants were likely to be deleterious (Table 1). Amino acid sequence alignment revealed that the c.1495A>G variant was highly conserved among the ten species included in our study (Figure 5).

**Table 1:** Bioinformatics software prediction of the deleteriousness of the serine active site domain-containing protein (*SERAC1*) variants found in this case study.

**Genetic variant**	**Protein change**	**Type of variant**	**Results**				
				**Mutation taster**	**Polyphen-2**	**PROVEAN**	**SIFT**
c.721_722del	p.Leu242fs	Deletion	Score	1.000	–	–	–
			Result	Disease causing	–	–	–
c.1495A>G	p.Met499Val	Missense	Score	0.999	1.000	−3.700	0.000
			Result	Disease causing	Probably damaging	Deleterious	Damaging

## Discussion

Previous studies have identified a causal relationship between *SERAC1* variants and MEGDEL syndrome (11–15). Approximately 70 cases of MEGDEL syndrome have been reported worldwide according to a report in 2020 (15). MEGDEL syndrome is serious, comprising an infantile-onset phenotype characterized by feeding problems, liver failure, spasticity, dystonia, hearing loss, truncal hypotonia, and premature death (16–18). The individual in this study only presented with one of the clinical characteristics of MEGDEL syndrome (3-MGA-uria), along with other symptoms not usually associated with this syndrome, including decreased myodynamia of both lower extremities, enhanced muscular tension and tendon reflexes, pyramidal tract injury, limp, and abnormal spinal and brain MRI; these symptoms were more similar to those of cHSP than MEGDEL syndrome. To the best of our knowledge, among more than 50 *SERAC1* variants included in HGMD, only one has so far been reported to be related to mild cHSP in a large family (9). The author introduced the milder cHSP caused by a novel *SERAC1* variant in a big family. Among the family members, none had a history of infantile feeding problems, liver failure, hearing loss, or truncal hypotonia. All were still able to walk several miles unaided aged between 10 and 20 years; the most severely affected of six siblings had signs of dystonia (9, 19). In brief, all these features were similar to those of the patient included in this study, which is only the second to report cHSP resulting from variants in *SERAC1*.

In our study, the proband was a 7-year-old female child with non-consanguineous, healthy Chinese parents. She had decreased myodynamia of both lower extremities, enhanced muscular tension and tendon reflexes, and pyramidal tract injury. Her brain MRI results showed bilateral basal ganglia alterations. These phenotypes were consistent with the clinical diagnosis of cHSP. A second MRI examination performed in June 2021 showed almost no change compared with the original MRI results. Clinical symptoms showed little change in 1 year since she was discharged from the hospital. The biochemical results showed 3-MGA-uria due to an elevated 3-MGA excretion in the urine. Our WES results, which covered more than 20,000 human nuclear genes and 37 human mitochondrial genes (including all 16,569 bases of mitochondrial DNA), found only *SERAC1* variants, with variants in any other genes related to cHSP. Given that there was one previous report of *SERAC1* variants resulting in cHSP, we concluded that the child in our study had juvenile-onset cHSP.

*SERAC1*, a novel cHSP gene located on chromosome 6q25.3, spans about 59 kb and contains 17 exons. The SERAC1 protein is 654 amino acids in length with a conserved serine-lipase domain (consensus lipase motif GxSxG); it is a member of the PGAP-like protein domain family (PFAM PF07819) (13, 19). SERAC1 is located within cells at the interface between the endoplasmic reticulum and the mitochondria and is involved in remodeling phosphatidylglycerol-34:1 (PG-34:1) to phosphatidylglycerol-36:1 (PG-36:1). This role is essential for both mitochondrial function and intracellular cholesterol trafficking. Previous researches have shown that *SERAC1* variants can increase the PG 34:1/PG 36:1 ratio (6, 9), resulting in mitochondrial dysfunction. In our study, the c.721_722delAG variant (p.Leu242fs) led to a frameshift during translation and a premature termination codon. As a result, this variant was suspected to be deleterious according to the 2015 American College of Medical Genetics and Genomics variant classification guidance. The missense variant c.1495A>G (p.Met499Val) was also predicted to be deleterious. However, the clinical significance of this variant is not yet clear. The two variants were found to be located in different domains; the c.1495A>G variant was highly conserved among ten species. The superimposed effect of the two variants may have resulted in a diseased phenotype in the individual in this study, but no clear relationship between *SERAC1* variants and phenotypes has been established (19), and the specific mechanism is also not clear. Therefore, further research is required to explore the potential mechanism.

The main finding of the study was that *SERAC1* variants not only can lead to MEGDEL syndrome but also can cause cHSP. However, the specific relationship between *SERAC1* variants and MEGDEL or cHSP is currently unclear. The mechanism by which *SERAC1* variants may cause these diseases is also unclear. Although both of the diseases are associated with elevated 3-MGA in urine and *SERAC1* variants, their clinical symptoms obviously differ. Therefore, functional validation experiments are required to explore the specific pathogenic mechanisms.

## Conclusion

To date, few cases of *SERAC1* variants associated with cHSP have been reported. Here, we reported a case of *SERAC1* variants resulting in cHSP, which is only the second to demonstrate this phenomenon. This case therefore adds to a number of known *SERAC1* variants and expands the phenotypic spectrum of SERAC1 deficiency. Thus, it will likely contribute to improved counseling and prevention of cHSP through a prenatal diagnosis of rare genetic metabolic diseases.

## Data Availability Statement

The original contributions presented in the study are included in the article/supplementary files, further inquiries can be directed to the corresponding author/s.

## Ethics Statement

Written informed consent was obtained from the individual(s), and minor(s)' legal guardian/next of kin, for the publication of any potentially identifiable images or data included in this article.

## Author Contributions

XZ and JZ collected the clinical information. JS and CC designed the experiment and performed the genetic analysis. CZ performed part of the data analyses. DY and SC wrote the manuscript and analyzed the data. FC and DL edited the final manuscript. All authors agreed to be accountable for and ensure any questions relating to the accuracy, integrity of this work, and read and approved the final manuscript.

## Funding

This work was supported by the National Natural Science Foundation of China (grant number 81771589), the Tianjin Science and Technology Plan Program (grant number 18ZXDBSY00170), and the Public Health and Technology Project of Tianjin (grant number ZC20120).

## Conflict of Interest

The authors declare that the research was conducted in the absence of any commercial or financial relationships that could be construed as a potential conflict of interest.

## Publisher's Note

All claims expressed in this article are solely those of the authors and do not necessarily represent those of their affiliated organizations, or those of the publisher, the editors and the reviewers. Any product that may be evaluated in this article, or claim that may be made by its manufacturer, is not guaranteed or endorsed by the publisher.
